# Prenatal Exposure to a Human Relevant Mixture of Endocrine-Disrupting Chemicals Affects Mandibular Development in Mice

**DOI:** 10.3390/ijms252212312

**Published:** 2024-11-16

**Authors:** Vagelis Rinotas, Antonios Stamatakis, Athanasios Stergiopoulos, Carl-Gustaf Bornehag, Joëlle Rüegg, Marietta Armaka, Efthymia Kitraki

**Affiliations:** 1 Institute for Fundamental Biomedical Research (IFBR), Biomedical Research Center “Al. Fleming”, 16672 Vari, Greece; 2 Biology-Biochemistry Laboratory, Faculty of Nursing, School of Health Sciences, National and Kapodistrian University of Athens (NKUA), 11527 Athens, Greece; astam@nurs.uoa.gr (A.S.);; 3 Department of Health Sciences, Karlstad University, 65188 Karlstad, Sweden; 4 Department of Environmental Medicine and Public Health, Icahn School of Medicine at Mount Sinai, New York, NY 10029, USA; 5 Department of Organismal Biology, Uppsala University, 75236 Uppsala, Sweden; 6 Laboratory of Basic Sciences, Faculty of Dentistry, School of Health Sciences, National and Kapodistrian University of Athens (NKUA), 11527 Athens, Greece

**Keywords:** EDCs, mice, mandible, micro-CT

## Abstract

Mandible is a bony structure of neuroectodermal origin with unique characteristics that support dentition and jaw movements. In the present study, we investigated the effects of gestational exposure to a mixture of endocrine-disrupting chemicals (EDCs) on mandibular growth in mice. The mixture under study (Mixture N1) has been associated with neurodevelopmental effects in both a human cohort and animal studies. Pregnant mice were exposed throughout gestation to 0.5× (times of pregnant women’s exposure levels), 10×, 100× and 500× of Mixture N1, or the vehicle, and the mandibles of the male offspring were studied in adulthood. Micro-CT analysis showed non-monotonic effects of Mixture N1 in the distances between specific mandibular landmarks and in the crown width of M1 molar, as well as changes in the mandibular bone characteristics. The alveolar bone volume was reduced, and the trabecular separation was increased in the 500× exposed mice. Bone volume in the condyle head was increased in all treated groups. Τhe Safranin-O-stained area of mature hypertrophic chondrocytes and the width of their zones were reduced in 0.5×, 10× and 100× exposed groups. This is the first indication that prenatal exposure to an epidemiologically defined EDC mixture, associated with neurodevelopmental impacts, can also affect mandibular growth in mammals.

## 1. Introduction

Endocrine-disrupting chemicals (EDCs) are substances of either natural or artificial origin that mimic or modify hormonal actions. EDCs are found in numerous widely used products including plastics, detergents, building materials, cosmetics, and pesticides. Organisms can be exposed to these hazards via inhalation and feeding, through the skin, the placenta, and the maternal milk during lactation [1]. Due to their rudimentary detoxification mechanisms, embryos and developing organisms are more vulnerable to the effects of these hormone-mimicking chemicals. Accumulating evidence shows that EDC exposures during critical time windows can modify the developmental process of certain systems, leading to long-lasting and even transgenerational aberrations [2].

The mandible or lower jaw differs from other bones in terms of biomechanics, morphology, physiology and morphogenesis. The mandible is the only mobile bone of the skull and has an important role in supporting dentition and in the outfit of the temporo-mandibular joint. Mandible formation is an early developmental process taking place between gestational weeks 7 and 14 in humans [3] and embryonic days E13.5 and 16.5 in mice [4]. During craniofacial development, neuroectoderm-derived neural crest cells interact with cells of ectoderm and paraxial mesoderm and migrate towards maxillary and mandibular prominences where they contribute to the formation of Meckel’s cartilage. The latter provides the template for mandibles, teeth, and other craniofacial structures [5]. These early cellular interactions require tight synchronization between cell proliferation and differentiation to avoid jaw aberrations that may permanently impact its shape and function. The process of oral bone formation and odontogenesis is sensitive to external interventions including chemicals and hormones. Rodents exposed in utero to ethanol [6], nicotine [7], or thyroxin [8] exhibit impaired mandible morphology at later life stages. Estrogens, apart from being significant contributors to the formation of the appendicular skeleton [9], participate in craniofacial bone development [10,11]. Evidence from estrogen receptor alpha (ERα) knockout mice (ERαKO C57BL/6) shows that estrogens, via ERα, mediate the maturation of mandibular condylar cartilage in males [12].

Consequently, estrogen-mimicking EDCs have been investigated for their effects on bone development. Perinatal exposure to Bisphenol A (BPA) or phthalates leads to impaired non-facial bone development in several species [13,14]. Regarding mandibular development, BPA and phthalates have been reported to affect the facial [15] and non-facial skeletal growth [16] in zebrafish. In rats, perinatal exposure to BPA disturbed amelogenesis in male offspring by altering estrogenic signaling [17,18]. Exposure of adult mice to di-(2-ethylhexyl) phthalate (DEHP) caused enamel defects in their continuously growing incisors [19]. In humans, the developmental impact of phenols and phthalates has been examined on total body (less head) bones, where maternal levels of these chemicals during pregnancy have been associated with decreased bone mineral density in their progeny [20,21,22].

Humans are exposed to chemical mixtures rather than single compounds [23]. In previous studies, we have composed human-relevant mixtures based on epidemiological data from the Swedish Environmental, Longitudinal, Mother and Child, Asthma and Allergy (SELMA) pregnancy cohort study [24]. Using weighted quantile sum (WQS) regression, we identified EDC mixtures measured in blood or urine of SELMA women around pregnancy week 10 associated with health outcomes in their children. Further analyses of these mixtures in different cell and animal models revealed significant effects on reproduction [25], metabolic growth [26,27,28] and neurodevelopment [28,29,30]. We have previously reported neurodevelopmental effects of Mixture N1, an EDC mixture associated with language delay at 30 months, by identifying that in utero exposure to Mixture N1 altered the brain transcriptome and epigenome and, consequently, the behavior of mice offspring in adulthood [29,30].

Considering the impact of Mixture N1 on neural tissue development, we aimed to examine whether this mixture has an effect on the development of the mouse mandible, a structure originating from cranial neural crest cells. For this reason, pregnant mice were exposed daily throughout gestation to Mixture N1 at doses of 0.5×, 10×, 100× and 500× (representing times of human serum concentrations measured or estimated in pregnant women of the SELMA cohort study) or the vehicle. We then performed micro-Computed Tomography (micro-CT) based analysis in the mandibles of adult male offspring to accurately quantify potential changes in mandibular morphometry and bone composition. Histological staining was applied to reveal the areas of mature hypertrophic chondrocytes in the condylar head.

## 2. Results

### 2.1. Mixture N1 Exposure Affects Mandibular Morphometry

**Figure 1:**
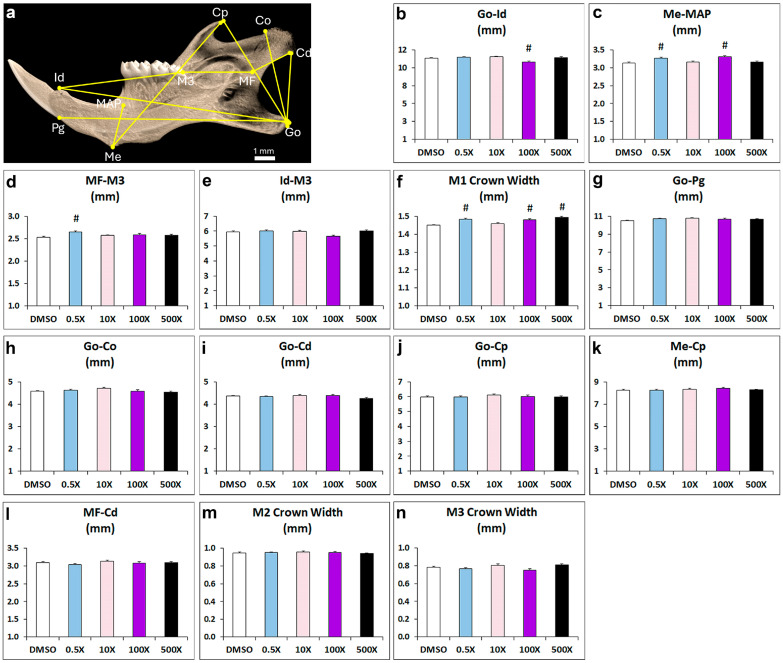
Morphometric analysis of distance measurements in mandibles from adult mice prenatally exposed to 0.5×, 10×, 100× and 500× of Mixture N1 or the vehicle (DMSO). Quantification of the measurements. Data represent estimated marginal means ± SEM. Generalized linear models (GLMs) were performed for statistical analysis followed by Bonferroni post hoc tests when appropriate. (**a**) Landmarks used in micro-CT analysis. (**b**) Distance from Go to Id was affected by the treatment (W4 = 26.548; *p* < 0.001) and was decreased in the 100× group vs. DMSO. (**c**) Distance from Me to MAP was affected by the treatment (W4 = 27.826; *p* < 0.001) and was increased in the groups 0.5× and 100×, compared to DMSO. (**d**) The distance from M3 molar to MF was affected by the treatment (W4 = 20.497; *p* < 0.001) and was increased in the 0.5× group. (**e**) The distance from M3 to Id was not significantly changed. (**f**) The Μ1 crown width was affected by the treatment (W4 = 66.279; *p* < 0.001) and was increased in the 0.5×, 100× and 500× groups. No significant effects were observed in the distances from Go to Pg (**g**), Go to Co (**h**), Go to Cd (**i**), Go to Cp (**j**), Me to Cp (**k**), MF to Cd (**l**), and in the crown width of molars M2 (**m**) and M3 (**n**). Go: gonion; Id: infradental; Me: menton; MAP: mandibular alveolar point; MF: mandibular foramen; Pg: pogonion; Co: dorsal condylar process; Cd: ventral condylar process; Cp: coronoid process; M: molar. # Statistically significant vs. DMSO group.

Prenatal exposure to Mixture N1 resulted in significant alterations in multiple landmarks of the offspring’s mandible in adulthood (Figure 1 and Table S1). A statistically significant reduction in the distance from gonion (Go) to infradental was evident in the 100× group vs. DMSO (Figure 1b). Furthermore, the width-related distance from menton to the mandibular alveolar point (MAP) was significantly increased in the groups 0.5× and 100×, compared to DMSO (Figure 1c). The distance from M3 molar to mandibular foramen (MF) was increased in the 0.5× group (Figure 1d), while M3 to infradental distance was not significantly changed (Figure 1e). Regarding the size of mandibles, a significant increase in the Μ1 crown width was prominent in the 0.5×, 100× and 500× groups compared to DMSO group (Figure 1f). No significant effects were observed in the distances between gonion (Go) to pogonion, Go to condyle dorsal part (Co), Go to condyle ventral part (Cd), Go to coronoid process (Cp), menton to Cp, MF to Cd, and in the crown width of molars M2 and M3 (Figure 1g–n).

Collectively, we observed increased distance from menton to MAP in mandibles of mice treated with 0.5× and 100×, but not in mice treated with 10× or 500× Mixture N1. The reduced distance from gonion to infradental was detected only in the 100× exposed mice. The width of the M1 molar crown was significantly increased in all experimental groups except for 10×. These findings suggest that prenatal exposure to increasing quantities of Mixture N1 leads to morphometric alternations in mandibles in a non-monotonic manner.

### 2.2. Mixture N1 Exposure Affects Mandibular Bone Composition

**Figure 2:**
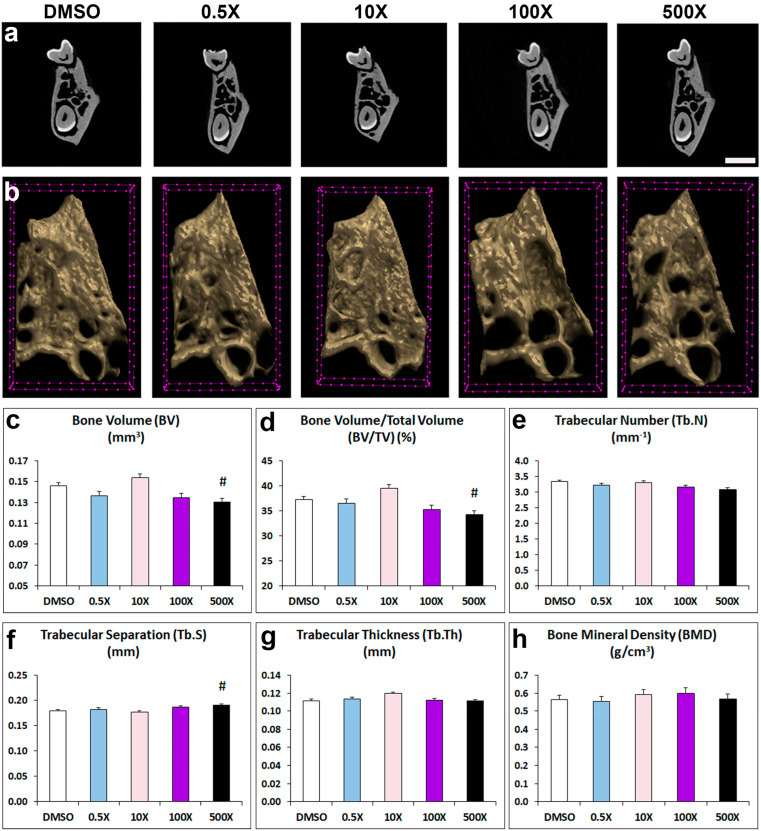
Representative 2D (**a**) and 3D (**b**) micro-CT images of trabecular alveolar bone from adult mice prenatally exposed to DMSO, 0.5×, 10×, 100× and 500× of Mixture N1. (**c**–**h**) Quantification of the measurements. Data represent estimated marginal means ± SEM. Generalized linear models (GLMs) were performed for statistical analysis followed by Bonferroni post hoc tests when appropriate. (**c**) BV was affected by the treatment (W4 = 27.966, *p* < 0.001) and was decreased in 500× vs. DMSO. (**d**) BV/TV was affected by the treatment (W4 = 29.316 *p* < 0.001) and was decreased in 500× vs. DMSO. (**f**) Tb.S was affected by the treatment (W4 = 18.371, *p* = 0.001) and was increased in 500× vs. DMSO. Trabecular number (Tb.N), (**e**), thickness (Tb.Th) (**g**) and bone mineral density (BMD) (**h**) did not differ significantly from DMSO group. # Statistically significant vs. DMSO group. Scale bars: 2a = 1 mm; 2b = 100 μm.

To explore the effects of Mixture N1 on adult structural bone parameters of the prenatally exposed offspring, we analyzed the microarchitecture of the alveolar bone, condylar cancellus bone and cortical bone (Figure 2 and Table S2). Mixture N1 treatment had significant effects on the alveolar trabecular bone of the 500× exposed mice. Specifically, alveolar bone volume (BV, Figure 2c) and bone volume fraction (BV/TV, Figure 2d) were decreased, while trabecular separation (Tb.S, Figure 2f) was increased compared to DMSO-treated offspring. No significant changes were observed in the other parameters of the alveolar bone: trabecular number (Tb.N, Figure 2e), thickness (Tb.Th Figure 2g), and bone mineral density, (BMD, Figure 2h) as compared to DMSO group.

**Figure 3:**
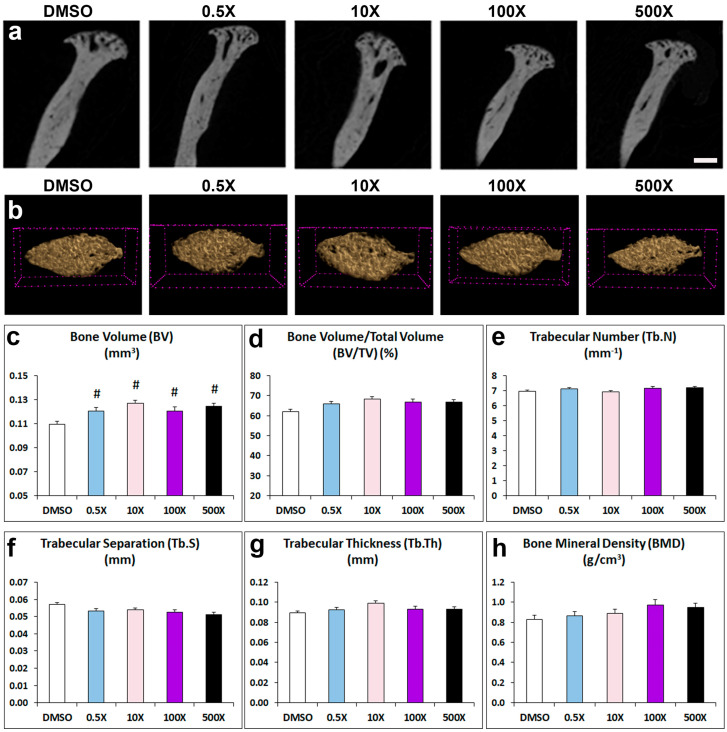
Representative 2D (**a**) and 3D (**b**) micro-CT images of cancellous bone in the condylar head from adult mice prenatally exposed to DMSO, 0.5×, 10×, 100× and 500× of Mixture N1. Quantification of the measurements (**c**–**h**). Data represent estimated marginal means ± SEM. Generalized linear models (GLMs) followed by Bonferroni post hoc tests when appropriate were performed for statistical analysis. (**c**) BV was affected by the treatment (W4 = 29.733, *p* < 0.001) and increased in all treated groups as compared to DMSO. # denotes statistical significance vs. DMSO treated group. Scale bars: 3a 400 μm; 3b 100 μm.

The micro-CT analysis of the cancellous bone in the condylar head showed a significant increase in the BV in all experimental groups treated with Mixture N1 compared to the DMSO group (Figure 3c). However, other important condylar bone parameters such as BV/TV, Tb.N, Tb.S, Tb.Th and BMD were not significantly affected by prenatal treatment with Mixture N1 (Figure 3d–h and Table S2).

**Figure 4:**
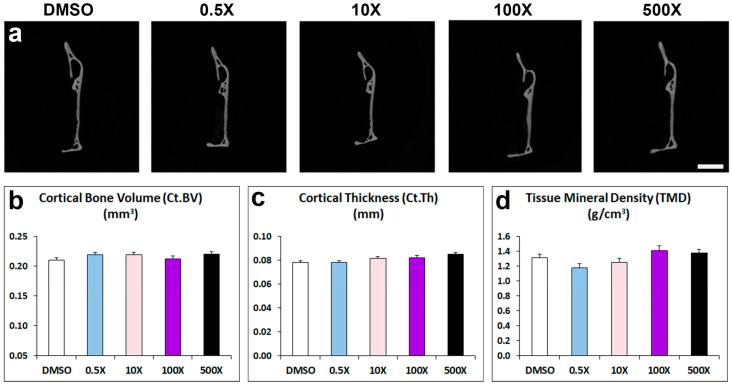
(**a**) Representative micro-CT images of cortical bone of the mandible from adult mice prenatally exposed to DMSO, 0.5×, 10×, 100× and 500× of Mixture N1. Quantification of the measurements for Ct.BV (**b**), Ct.Th (**c**) and TMD (**d**). Data represent estimated marginal means ± SEM. Generalized linear models (GLMs) were performed for statistical analysis. Scale bar: 1 mm.

In contrast to the detected changes in the alveolar and condylar bone, the architecture of mandibular cortical bone of adult male offspring prenatally exposed to Mixture N1 was not affected in terms of cortical bone volume, thickness and tissue mineral density (Figure 4 and Table S2).

### 2.3. Mixture N1 Exposure Affects Condylar Cartilage

**Figure 5:**
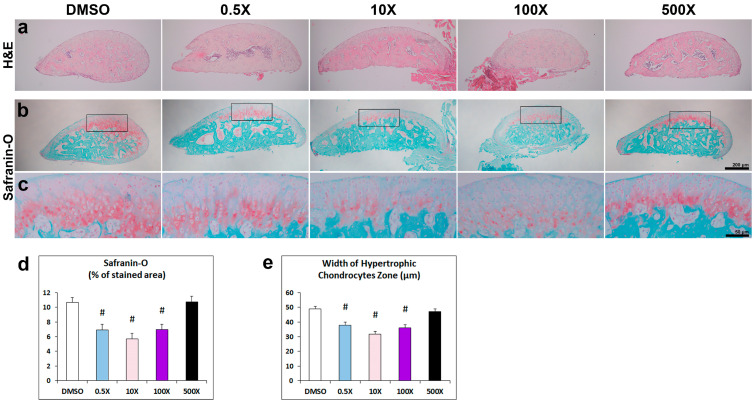
(**a**–**c**) Representative photomicrographs of the condylar head from adult mice prenatally exposed to DMSO, 0.5×, 10×, 100× and 500× of Mixture N1, stained with H&E (**a**) and Safranin-O-red/Methylene green (**b**,**c**). Higher magnification images in (**c**) correspond to the inset area of respective figures in (**b**). (**d**) Quantification of % condyle head area stained with Safranin-O. The Safranin-O-stained area was modified by the Mixture exposure (W4 = 39.939; *p* < 0.001). The % of Safranin-stained area was significantly reduced vs. DMSO in the groups of 0.5× (*p* = 0.002), 10× (*p* < 0.001) and 100× (*p* = 0.002), but not in the 500× group (*p* = 1.000). (**e**) The width of the hypertrophic chondrocytes’ zone was modified by the Mixture exposure (W4 = 62.003; *p* < 0.001). It was significantly reduced vs. DMSO in the groups of 0.5×, 10× and 100× (*p* < 0.001 for all). Data represent estimated marginal means ± SEM. Generalized linear model (GLM) and Bonferroni post hoc test were performed for statistical analysis. # denotes statistical significance vs. DMSO treated group. Scale bars: 200 μm in (**a**,**b**); 50 μm in (**c**).

Following the detected changes in the cancellous bone of the condyle, we pursued to detect corresponding histological alterations of the condylar head. Hematoxylin and Eosin (H&E) staining provided a first indication of the characteristic zonal cartilage organization of this area (Figure 5a). Safranin-O/Methylene green was utilized to stain the glycosaminoglycans- and proteoglycans-positive hypertrophic chondrocytes (Figure 5b,c and Figure S1). We detected a significant decrease in Safranin-O-stained area in the 0.5×, 10× and 100× groups (Figure 5d). The extent of the hypertrophic chondrocytes’ zone was further examined by measuring the width of their area along the condylar head, based on their morphology. Statistical evaluation of these data further showed the decreased extent of these cells in the 0.5×, 10× and 100× groups (Figure 5e).

## 3. Discussion

Mixture N1 composition (DEP, DBP, DBzP, DIDP/DPHP) BPA, TCP, 3-PBA and p,p′-DDE), was defined by associating maternal levels of exposure to chemicals during pregnancy with the neurodevelopmental progress of their children in the SELMA study [24]. We have previously demonstrated the significant impact of Mixture N1 on neurodevelopment upon gestational exposure in mice [29,30]. The present study showed a developmental effect of Mixture N1 on structural parameters of the murine mandible, which exhibits a cranial neural crest origin. Specifically, in utero N1 exposure modified the distances between several landmarks of the mandible and the crown width of the M1 molar, it reduced the trabecular alveolar bone volume (BV), whereas it increased the BV in the cancellous bone and altered the composition of the chondrocyte zone of the condylar head.

Mandibular shape and size are critical for the proper functioning of the temporomandibular junction and mastication. Consequently, changes in shape and size may lead to malocclusion. Our results showed altered distances between cephalometric landmarks of mandibular length (gonion to infradental, infradental to M3 molar and mandibular foramen to M3) and width (menton to MAP) in the Mixture-N1-exposed mice. This suggests a potential deformity of mandibular shape that could have influenced the increase in the width of the M1 molar, also witnessed in several of the experimental groups. Many of the detected effects exhibited a non-monotonic pattern that has often been reported to characterize the action of EDCs [31,32].

Mandibular composition provides an ideal microenvironment to support dentition and at the same time to endure multidirectional forces during chewing. Accordingly, the ramus and the angular process consist of cortical bone, while the alveolar region that hosts the teeth, and the condylar process consists of trabecular bone. In our study, the impact of Mixture N1 was detected in the trabecular areas of the alveolar bone and the condylar head. The reduced alveolar bone volume in teeth sockets contributes to periodontium loosening and potential teeth loss. Although studies linking phthalates with periodontitis are scarce, a recent epidemiological study associated urinary phthalate metabolites with increased odds of periodontitis in adult subjects [33]. The differential impact of Mixture N1 on the alveolar bone volume fraction (reduced) and the cancellous bone of the condyle (increased) may reflect the different ossification modes of these structures. The alveolar bone at the intermediate part of the mandible (in between the molar roots) is formed by intramembranous ossification, while the condyle follows endochondral ossification [34]. The condylar cartilage serves as a site of condylar growth through the hypertrophic chondrocytes that transdifferentiate into osteoblasts in the deeper layers of the condyle [35].

Our results showed a reduced area of hypertrophic chondrocytes in the condyle of groups exposed to 0.5×, 10× and 100× Mixture N1. It has been shown that condylar cartilage growth in both sexes of mice is supported by gonadal steroids, especially by estrogens via ERa [12,36]. It is also known that components of Mixture N1 such as BPA and phthalates interfere with estrogen signaling [37,38]. Although direct comparisons cannot be made between the effects of single EDCs and EDC mixtures, evidence from mice perinatally exposed to BPA (200 μg BPA/kg BW) shows reduced bone volume in the trabecular femoral bones of adult male offspring [39]. In our study, prenatal exposure to a lower dose of BPA (74.99 μg/kg/kg BW in the 500× group of Mixture N1 [26]) had similar effects in the mandibular alveolar bone. Furthermore, BPA could have also acted as an androgen receptor (AR) antagonist [40], counteracting the protective effects of androgens on trabecular bone mass exerted via AR signaling in osteoblasts [36]. Nevertheless, the effects of EDC mixtures cannot be directly extrapolated to those of single compounds due to the potential mutual interactions among the contributing chemicals [41]. These interactions could account for the lack of effects on cortical bone in our study, although it has been shown that developmental BPA exposure increases the cortical thickness of femoral bones in adult male rat offspring [42] that exhibit the same ossification pattern with mandibular cortical bone.

### Strengths and Limitations

To our knowledge, this is the first study to report impaired development of mandibular components in a mammalian model upon prenatal exposure to a human-relevant EDC mixture. Further studies are required to delineate the mechanisms of in utero effects of EDC mixtures in oral osteogenesis and dentition, since developmental alterations in mandible/condyle growth may lead to inadequate morphology and function in later life, leading to malocclusion and other complications [43]. Given that mandibular alterations were detected in mice offspring even at low doses corresponding to the 0.5× and 10× (times) of the exposure concentrations measured in pregnant women of the SELMA cohort study, it would be relevant to include dental examinations in the follow-up of children from such cohorts.

## 4. Conclusions

Our results provide the first evidence regarding the impact of gestational exposure to several doses of an epidemiologically defined EDC mixture (Mixture N1) in the mandible of adult male mouse offspring. The detected effects concern modified distances between specific landmarks of the mandibular skeleton, changes in the alveolar and condylar mandibular bone composition, as well as in the extent of hypertrophic chondrocytes in the condylar head. The effects exhibited a non-monotonic pattern. More research is required to reveal the potential mechanisms of these effects, as well as the mixture’s impact on the appendicular skeleton.

## 5. Methodology

### 5.1. Mixture N1

A detailed description of the establishment of Mixture N1 can be found in our previous publications [29,30]. In brief, the urine and serum levels of 26 suspected or known EDCs or their metabolites detected in 2354 women of the SELMA study at median pregnancy week 10 were subjected to weighted quantile sum (WQS) regression analysis to select those chemicals that were associated with language delay in children at 2.5 years of age. The daily intake (DI) of the selected chemicals, the plasma concentrations from the DI estimates, and the geometric means of both urinary and serum of these chemicals were then used to determine their mixing proportions. The composition of Mixture N1 is shown in Table 1. Phthalates’ active monoesters were used to prepare the mixture for mice. The chemicals were purchased from the following sources: Bisphenol A (BPA; 99%), Dimethylsulfoxide (DMSO; 99.9%), Monobenzyl phthalate (MBzP; 98%), 3-Phenoxybenzoic acid (3-PBA; 98%), and Trichloropyridinol (TCP; 99%)were obtained from Sigma-Aldrich Inc. (St. Louis, MO, USA). Monoethyl phthalate (MEP; 98%)and mono-iso-decyl phthalate (MiDP; 98%) were obtained from Toronto Research Chemicals (North York, ON, Canada).Mono-(2-propylheptyl)-phthalate(MPHP, 99%) were synthesized by Novandi Chemistry AB, Södertälje, Sweden. Monobutyl phthalate (MBP; 95%) was purchased from TCI, Tokyo Chemical Industry Co., Ltd. (Tokyo, Japan). pp′DDE was synthesized in house by Åke Bergman from DDT. For MIX N1, 1 M solutions in DMSO were prepared of each of the chemicals: BPA, MEP, MBP, BBzP, MIDP, MPHP, 3-PBA, and TCP. A 50/50 mixture of MIDP and MPHP was used [29].

### 5.2. Experimental Protocol and Tissue Preparation

In the present study, we examined the effect of prenatal exposure to Mixture N1 on the mandibles of three-month-old male offspring. The animal tissues and data were collected in a previous study for Mixture N1 [29]. The study’s protocol was approved by the Ethical Committee of the Prefecture of Attica-Veterinary Department (#4783) and performed in accordance with the European Communities Council Directive of 22 September 2010 (2010/63/EU). In brief, two-month-old C57/BL6 mice, purchased from the Hellenic Pasteur Institute (Athens, Greece), were used for breeding. The animals were housed under standard conditions of temperature and illumination and were offered a phytoestrogen-deficient pellet food (Altromin 1324P, Lage, Germany) and tap water ad libitum. From gestational day 1 to parturition, pregnant mice received daily via food the vehicle (Dimethylsulfoxide, DMSO) or the N1 mixture at doses of 0.5×, 10×, 100× and 500× hsc (*times of human serum concentration* represents the exposure concentrations relative to the geometric mean of the concentrations measured in pregnant women of the SELMA cohort study). Accordingly, the daily exposure of pregnant dams throughout pregnancy was 0.001, 0.22, 2.2 and 11 mg/kg bw of Mixture N1, respectively. Working solutions of different doses were prepared using DMSO 99.9% purity, Sigma-Aldrich Inc. (St. Louis, MO, USA). For all pregnant mice, DMSO intake did not exceed 0.25 μL/gr bw/day [29], which is considered a non-toxic exposure. Offspring were euthanized at three months of age under isoflurane anesthesia. The mandibles were removed and fixed in neutral formalin. One hemimandible was dehydrated and kept for micro-CT analysis. The respective condyles from the other half were decalcified in 10% EDTA solution, washed in tap water, dehydrated, and embedded in paraffin. The number of samples per group used in the analysis was: DMSO: n = 10, 0.5×: n = 8, 10×: n = 10, 100×: n = 7, 500×: n = 11.

### 5.3. Micro-CT Analysis

The microarchitecture of the mandibular bones was evaluated using a high-resolution SkyScan1172 microcomputed tomography (mCT) imaging system (Bruker, Aartselaar, Belgium). Images were acquired at 60 KeV, 100 µA, pixel size 10 μm, with a 0.5 mm aluminum filter. Two-dimensional reconstruction images were generated using Nrecon software (version 1.7.4.6) (Bruker) and analyzed using Ctan software (version 1.20.8) while 3D reconstructed images of alveolar bone were generated using Ctvox software (version 3.3.1) (Bruker). Morphometric measurements were performed with Data Viewer software (version 1.6.0) (Bruker micro-CT). Analyses were performed between the following reference points: gonion (Go) to dorsal point of the condylar process (Co); Go to ventral point of the condylar process (Cd); Go to coronoid process (Cp); Go to the most antero-dorsal point on mandibular symphysis (pogonion, Pg); Go to the most postero-dorsal point on mandibular symphysis (infradental, Id); menton (Me) to mandibular alveolar point (MAP); Me to Cp, mandibular foramen (MF) to Co; MF to M3 molar; Id to the most posterior part of M3 molar (Figure 1a). The crown width of M1, M2 and M3 molars was also measured.

Alveolar, trabecular, and cortical bones were analyzed from transaxial sections, respectively, between the roots of the first molar, at the mandibular condyle, and at the posterior edge of the ramus. The parameters of bone volume (BV, mm^3^), tissue volume (TV, mm^3^), bone volume fraction (BV/TV, %), trabecular number (Tb.N, mm^−1^), trabecular separation (Tb.S, mm), trabecular thickness (Tb.Th, mm) and bone mineral density (BMD, g/cm^3^) were measured for alveolar trabecular bone and condyle head. Cortical bone was assessed through cortical bone volume (Ct.BV, mm^3^), tissue volume (TV, mm^3^), cortical thickness (Ct.Th, mm), and tissue mineral density (TMD, g/cm^3^).

### 5.4. Histomorphometry of Condylar Head

The basic histology of the condylar head was examined in 4 μm paraffin sections (Leica RM 2125 microtome, Leica Microsystems, Wetzlar, Germany) stained with Hematoxylin and Eosin (H&E). Safranin-O/Methylene green staining was used to locate the zone of hypertrophic chondrocytes in the condylar head. Digital images of stained sections, corresponding to the mid-coronal part of the condylar head, were obtained by optical Microscope (Eclipse E400, Nikon, Tokyo, Japan) and the stained areas were quantified as % of the total condylar head area using ImageJ Software (v. 1.54f, 2023, NIH Image, National Institutes of Health, Bethesda, MD, USA) by two independent observers blindly. Animal samples used in quantification of staining: DMSO: n = 6; 0.5×: n = 5; 10×: n = 4; 100×: n = 6; 500×: n = 4. For each sample, an average of 3–5 sections was used in the analysis. In addition, the area width of hypertrophic chondrocytes was measured in the same samples based on the morphology of these cells. Specifically, for each available section from each animal/group, we performed 8–12 (usually 10) vertical measurements in the hypertrophic chondrocytes’ area, in most cases following the palisades formed by the hypertrophic chondrocytes. We used a systematic-random sampling method starting at a random point at the left end of the zone and measuring every 50 μm (lateral step), to cover the whole length of the zone.

### 5.5. Statistical Evaluation

Data were analyzed by generalized linear models (GLMs), with the dose of Mixture N1 (treatment) as the predictor factor and the treatment (litter) as a build nested predictor factor (IBM Statistical Package for the Social Sciences (SPSS) v. 21). In the case of statistically significant dose effects, Bonferroni post hoc tests were used to determine specific group differences compared to the DMSO-treated group. To control for type I error due to multiple statistical comparisons, an adjusted *p*-value threshold (*p* < 0.0015) has been calculated dividing *p* = 0.05 by the total number of statistical comparisons performed.

## Figures and Tables

**Table 1:** Composition of Mixture N1.

Compound	GM (nmol/mL)	Mixing Percentages (% of Urine + Serum)
**DEP**	0.03204	44.80
**DBP**	0.02855	39.92
**BBzP**	0.00568	7.94
**DIDP/DPHP**	0.00352	4.92
**BPA**	0.00047	0.66
**TCP**	0.00056	0.78
**3-PBA**	0.00011	0.15
**p,p′-DDE**	0.00059	0.82
**Total**	0.0715	100

Geometric means (GM) in pregnant women in SELMA for chemical compounds in the urine or serum and their mixing percentages. DEP: Di-ethyl phthalate, DBP: Di-butyl phthalate, BBzP: Benzyl butyl phthalate, DIDP: Diisodecyl phthalate, DPHP: Di(2-propylheptyl) phthalate, Bis(2-propylheptyl) benzene-1,2-dicarboxylate and di(propylheptyl) orthophthalate, BPA: Bisphenol A, TCP: Trichloropyridinol, 3-PBA: 3-Phenoxybenzoic acid, p,p′-DDE: 72-55-9; Dichloro diphenyl dichloro ethylene.

## Data Availability

The original datasets are presented in the article and Supplementary Materials. Further inquiries can be directed to the corresponding author.

## Supplementary Materials

The following supporting information can be downloaded at: https://www.mdpi.com/article/10.3390/ijms252212312/s1.
