# Supplementary material for: Prenatal Exposure to a Human Relevant Mixture of Endocrine-Disrupting Chemicals Affects Mandibular Development in Mice
Source: Int J Mol Sci. 2024 Nov 16;25(22):12312. doi: 10.3390/ijms252212312 (PMC11594603; doi:10.3390/ijms252212312)
Supplement: Supplementary file 1 [file ijms-25-12312-s001.zip › ijms-3225940-supplementary.pdf]

Supplemental Materials

"**Prenatal exposure to a human relevant mixture of EDCs affects mandibular development in mice**"

Vagelis Rinotas, Antonios Stamatakis, Athanasios Stergiopoulos, Carl Gustaf Bornehag, Joëlle Rüegg, Marietta Armaka* and Efthymia Kitraki* (*corresponding authors).

Table S1: Micro-CT measured landmark distances in mandibles from adult male mice in utero exposed to different doses of Mixture N1 or the vehicle (DMSO).

	DMSO	0.5X	10X	100X	500X
**Go-Co** (mm)	4.575 ± 0.036 N=10	4.621±0.041 N=8	4.715±0.039 N=10	4.591±0.048 N=7	4.545±0.038 N=10
**Go-Cd** (mm)	4.363±0.032 N=10	4.339±0.036 N=8	4.395±0.035 N=10	4.382±0.043 N=7	4.265±0.034 N=10
**Go-Cp** (mm)	5.970±0.064 N=10	5.980±0.078 N=7	6.105±0.075 N=8	6.016±0.087 N=6	5.976±0.071 N=9
**Go-Pg** (mm)	10.520±0.052 N=10	10.723±0.067 N=6	10.785±0.060 N=9	10.650±0.095 N=3	10.665±0.057 N=10
**Go-Id** (mm)	10.946±0.074 N=10	11.061±0.084 N=8, *p=1.000*	11.116±0.081 N=10, *p=0.721*	**10.519±0.098#** N=7 *p=0.004*	11.048±0.081 N=10, *p=1.000*
**Me-MAP** (mm)	3.136±0.023 N=10	**3.261±0.027#** N=8 *p=0.004*	3.152±0.026 N=10, *p=1.000*	**3.301±0.031#** N=7 *p<0.001*	3.163±0.024 N=11, *p=1.000*
**Me-Cp** (mm)	8.257±0.063 N=10	8.251±0.078 N=8	8.342±0.075 N=10	8.417±0.087 N=6	8.271±0.071 N=9
**MF-Cd** (mm)	3.095±0.028 N=10	3.029±0.031 N=8	3.132±0.030 N=10	3.073±0.037 N=7	3.091±0.029 N=10
**MF-M3** (mm)	2.530±0.018 N=10	**2.649±0.020#** N=8 *p<0.001*	2.570±0.019 N=10, *p=0.501*	2.590±0.023 N=7, *p=0.277*	2.573±0.018 N=11, *p=0.430*
**Id-M3** (mm)	5.950±0.061 N=10	6.000±0.069 N=8	5.994±0.067 N=10	5.642±0.081 N=7	6.026±0.067 N=10
**M1** (mm)	1.450±0.004 N=10	**1.483±0.005#** N=8 *p<0.001*	1.458±0.005 N=10, *p=0.409*	**1.480±0.006#** N=7 *p<0.001*	**1.494±0.004#** N=11 *p<0.001*
**M2** (mm)	0.946±0.007 N=10	0.948±0.008 N=8	0.956±0.008 N=10	0.950±0.009 N=7	0.939±0.007 N=11
**M3** (mm)	0.779±0.013 N=10	0.763±0.015 N=8	0.804±0.015 N=10	0.747±0.018 N=7	0.807±0.014 N=11

Co: dorsal condylar process; Cd: ventral condylar process; Cp: Coronoid process; Go: Gonion; Id: Infradental; M: Molar crown width; MAP: Mandibular Alveolar Point; Me: Menton; MF: Mandibular Foramen; Pg: Pogonion.

Numbers represent estimated marginal means +/- SEM. N: number of samples analyzed per group; p: p-values. # Statistically significant vs. DMSO-treated group.

Table S2: Micro-CT measured mandibular alveolar, condylar and cortical bone components in samples of adult mice in utero exposed to different doses of Mixture N1 or the vehicle (DMSO).

	DMSO	0.5X	10X	100X	500X
**Alveolar bone**					
**BV** (mm^3)	0.1458±0.003<br>N=9	0.1367±0.0037<br>N=8, *p=0.334*	0.1538±0.0036<br>N=10, *p=0.411*	0.1346±0.0043<br>N=7, *p=0.237*	**0.1304±0.0034#**<br>N=11, *p=0.008*
**TV** (mm^3)	0.3922±0.003<br>N=9	0.3742±0.0039<br>N=8	0.3897±0.0038<br>N=10	0.3824±0.0046<br>N=7	0.3798±0.0035<br>N=11
**BV/TV** (%)	37.263±0.675<br>N=9	36.5771±0.7689<br>N=8, *p=0.841*	39.4902±0.7409<br>N=9, *p=0.158*	35.2259±0.8986<br>N=7, *p=0.280*	**34.3095±0.6968#**<br>N=11, *p=0.019*
**BMD** (g/cm^3)	0.5630±0.025<br>N=9	0.5529±0.0287<br>N=8	0.5933±0.0277<br>N=10	0.5977±0.0336<br>N=7	0.5678±0.0260<br>N=11
**Tb.Th** (mm)	0.1117±0.001<br>N=9	0.1137±0.0018<br>N=8	0.1195±0.0017<br>N=10	0.1118±0.0021<br>N=7	0.1114±0.0016<br>N=11
**Tb.N** (1/mm)	3.3378±0.049<br>N=9	3.2243±0.0564<br>N=8	3.3082±0.0543<br>N=10	3.1655±0.0659<br>N=7	2.9867±0.0511<br>N=11
**Tb.S** (mm)	0.1788±0.002<br>N=9	0.1820±0.0026<br>N=8, *p=1.000*	0.1769±0.0025<br>N=10, *p=1.000*	0.1864±0.0030<br>N=7, *p=0.281*	**0.1897±0.0023#**<br>N=11, *p=0.009*
**Condylar bone**					
**BV** (mm^3)	0.1094±0.002<br>N=10	**0.1206±0.0027#**<br>N=8, *p=0.017*	**0.1270±0.0026#**<br>N=10, *p<0.001*	**0.1207±0.0032#**<br>N=7, *p=0.033*	**0.1244±0.0025#**<br>N=11, *p<0.001*
**TV** (mm^3)	0.1758±0.002<br>N=10	0.1835±0.0033<br>N=8	0.1856±0.031<br>N=10	0.1814±0.0038<br>N=7	0.1854± 0.0030<br>N=11
**BV/TV**	62.150±1.126<br>N=10	65.9046±1.2820<br>N=8	68.3469±1.2354<br>N=10	66.7517±1.4984<br>N=7	66.8714±1.1869<br>N=11
**BMD** (g/cm^3)	0.8307±0.037<br>N=10	0.8620±0.0421<br>N=8	0.8900±0.0406<br>N=10	0.9751±0.0493<br>N=7	0.9504±0.0390<br>N=11
**Tb.Th** (mm)	0.0895±0.002<br>N=10	0.0927±0.0022<br>N=8	0.0993±0.0022<br>N=10	0.0934±0.0026<br>N=7	0.0932±0.0021<br>N=11
**Tb.S** (mm)	0.0571±0.001<br>N=10	0.0534±0.0013<br>N=8	0.0538±0.0012<br>N=10	0.0525±0.0015<br>N=7	0.0513±0.0012<br>N=11
**Tb.N** (1/mm)	6.9675±0.085<br>N=10	7.1120±0.0967<br>N=8	6.9145±0.0932<br>N=10	7.1600±0.1130<br>N=7	7.2122±0.0895<br>N=11
**Cortical bone**					
**Ct.BV** (mm^3)	0.2104±0.003<br>N=9	0.2189±0.0040<br>N=8	0.2195±0.0038<br>N=9	0.2121±0.0047<br>N=7	0.2201±0.0036<br>N=9
**TV** (mm^3)	0.2215±0.003<br>N=9	0.2305±0.0045<br>N=8	0.2277±0.0043<br>N=9	0.2208±0.0052<br>N=7	0.2276±0.0040<br>N=9
**TMD** (g/cm^3)	1.3108±0.0464<br>N=10	1.1776±0.0528<br>N=8	1.2504±0.0509<br>N=9	1.4093±0.0618<br>N=7	1.3771±0.0479<br>N=9
**Ct.Th** (mm)	0.0779±0.001<br>N=10	0.0780±0.0018<br>N=8	0.0814±0.0017<br>N=9	0.0819±0.0021<br>N=7	0.0848±0.0016<br>N=9

Numbers represent estimated marginal means +/- SEM. N: number of samples analyzed per group. BV, bone volume; TV, tissue volume; TMD, tissue mineral density; BMD, bone mineral density; Tb.N, trabecular number; Tb.Th, trabecular_Thickness; Tb.Sp, trabecular separation. # Statistically significant vs. DMSO.
